# Impact of a sugar-sweetened beverage tax on sugar producers in Ukraine

**DOI:** 10.1093/eurpub/ckad083

**Published:** 2023-05-18

**Authors:** Kate L Mandeville, Oleg Nivievskyi, Roman Neyter, Pavlo Martyshev, Volodymyr Vakhitov, Bethany Warren, Olena Doroshenko

**Affiliations:** Health, Nutrition, and Population Global Practice, World Bank, Washington, DC, USA; Department of Global Health and Development, London School of Hygiene and Tropical Medicine, London, UK; Kyiv School of Economics, Kyiv, Ukraine; University of Göttingen, Göttingen, Germany; Kyiv School of Economics, Kyiv, Ukraine; Kyiv School of Economics, Kyiv, Ukraine; Kyiv School of Economics, Kyiv, Ukraine; Health, Nutrition, and Population Global Practice, World Bank, Washington, DC, USA; Resolve to Save Lives, New York, NY, USA; Health, Nutrition, and Population Global Practice, World Bank, Washington, DC, USA

## Abstract

Taxes on sugar-sweetened beverages can promote health and raise revenue. Whether these taxes negatively impact domestic sugar producers, an argument often made by opponents, is understudied. We extended a simulation model based on a uniform specific volume-based tax of UAH 4/L in Ukraine. We estimated best- and worst-case scenarios for reductions in domestic sugar demand to be 162 and 23 000 metric tons. This is at worst ∼0.5% of current exports, meaning decreases in domestic demand could easily be absorbed by export markets given export trends. Due to highly protectionist sugar sector policy, sugar producers would not be able to fully substitute domestic sales revenues through increased export revenues, but the worst-case revenue gap was <0.5% of total sectoral output in recent years. Overall, introducing a tax on sugar-sweetened beverages in Ukraine is likely to have a very limited impact on domestic sugar producers.

## Introduction

Sugar-sweetened beverage (SSB) taxes have been gaining momentum globally as countries grapple with increases in obesity and other non-communicable diseases, as well as the need to raise additional government revenue.^1^ Opposition to SSB taxes can quickly mobilize using a variety of arguments, including that they negatively impact business and employment. These arguments often do not hold up under independent examination.^2^ In sugar-producing countries, the impact on domestic sugar production can be a key source of opposition and undermine political will to introduce or maintain such taxes. For example, in South Africa, concerns over job losses for sugar farmers and others in the sugar value chain gained traction given the country’s high unemployment rate.^3^ Yet, there is little evidence on the impact of SSB taxes on domestic sugar production in the literature. One analysis for Australia estimated that a SSB tax would reduce demand for domestically produced sugar by just one percent, meaning that slightly more sugar would need to be exported rather than sold domestically.^4^ Moreover, decreased demand due to an SSB tax may be an opportunity for the sugar industry to restructure, diversify into alternative crops or market outlets, and explore alternative segments (e.g. sweeteners) given growing anti-sugar consumer preferences.

Ukraine is a sugar-producing country, drawing on favorable conditions for sugar beet farming. Due to its historical importance, the sugar industry has been greatly protected from international competition in Ukraine,^5^ with imports highly restricted by a tariff-rate quota system that ensures up to 50% higher domestic sugar prices than reference market prices. In periods of favorable sugar beet harvests, industry has exported sugar surpluses abroad. Despite high protection, the sugar industry has been shrinking and now plays only a marginal role in Ukraine’s economy. Overall, domestic consumption has been decreasing for over 20 years and is now consistently lower than domestic production.^6^ Despite this, dietary risks are a leading contributor to death and disability. In support of the government’s Healthy Ukraine strategy to address these issues, we examined the potential impact of a SSB tax on sugar producers in Ukraine and report it here as an example for countries to consider as they explore SSB taxes.

## Methods

This work was undertaken as part of a collaboration to examine the potential of a SSB tax in Ukraine. We extended a simulation model by the World Health Organization (WHO) that estimated the effect of a SSB tax on the sales volumes of taxed and untaxed SSB.^7^ This model was based on a uniform specific volume-based tax of UAH 4/L (approximating a 20% increase in retail price) and structured as a category-level static model with six SSB categories: carbonates, (packaged) juices, (bottled) water, energy and sports drinks, (packaged) coffee and tea, and concentrates. This means effects can be estimated based on which SSBs the tax is applied to, as SSB taxes often exclude certain categories (e.g. bottled water). A range of scenarios were modeled, including sensitivity analysis to assess the effect of the results of using different excluded SSBs, price elasticities and pass-through rates.

We estimated current sugar demand from the SSB industry in Ukraine, by applying average sugar content for each SSB category to Euromonitor data on volume of SSBs sold in Ukraine in 2019.^8^ Due to a lack of data on sugar content of different SSBs in Ukraine, mean total sugar content values were taken from a 2015 cross-sectional survey of sugar content of pre-packaged foods and drinks in Slovenia.^9^ We used the most appropriate available value for each SSB category, as follows: sodas (carbonates), fruit and vegetable juices (juices), waters (water), electrolyte drinks (energy and sports drinks), coffee and tea (coffee and tea) and cordial (concentrates).

We then estimated both best- and worst-case scenarios for reductions in sugar demand from the tax. We selected the scenarios in the WHO analysis that resulted in the smallest (−1%) and largest (−21%) reduction in overall sales volumes of taxed SSB. Drawing on the percent change in volume for each individual SSB category from the WHO analysis and the sugar content values as above, we estimated the change in volume in liters and resulting change in sugar demand in metric tons (1000 kg). The best- and worst-case scenarios represent a range in which the true value is likely to be included. As sugar is sold at a higher price domestically than exports, we then estimated the revenue loss from any change in demand as a percentage of the total sugar sector output in in 2018 and 2019, using price and revenue data from the State Statistics Committee of Ukraine.^10^

## Results

The SSB industry in Ukraine created an estimated 164141 metric tons of sugar demand in 2019, the majority from carbonates (table 1). This represented 9.4% of beet sugar production and 11.3% of total sugar consumption in Ukraine in 2018/2019.^6^

**Table 1:** Best- and worst-case scenarios for reductions in sugar demand as a result of implementation of a uniform specific volume-based SSB tax of UAH 4/L in Ukraine

	Estimated baseline sugar demand			Best-case scenario^a^			Worst-case scenario^b^		
SSB category	**Volume sold in 2019^c^** *L, thousands*	**Sugar content^d^** *grams per L*	**Sugar demand^e^** *metric tons*	**Change in volume^f^** *percent*	**Change in volume** *L, thousands*	**Change in sugar demand** *metric tons*	**Change in volume^e^** *percent*	**Change in volume** *L, thousands*	**Change in sugar demand** *metric tons*
**Carbonates**	1 511379	77	116 376	−2	−30228	−2328	−21	−317390	−24 439
**Juices**	339 633	95	32 265	5	16982	1613	9	30567	2904
**Bottled water**	2 095 043	0^g^	314 256	15	0	0	9	188554	0
**Energy & sports drinks**	46145	99	4568	9	4153	411	−6	−2769	−274
**Packaged coffee & tea**	45 550	235	10704	2	911	214	−15	−6833	−1606
**Concentrates**	713	319	227	−32	−228	−73	9	64	20
**Total**	4 038 463		164141		−8410	−162		−296 360	**−**23394

a Best-case scenario excluded bottled water and assumed the tax will be fully passed onto consumers (without any under- or over-shifting). It also used a price elasticity of −0.55, cross-price elasticities of 0.1, and income elasticity of 0.3.

b Worst-case scenario excluded water, juices, and concentrates and assumes extreme pass-through (over-shifting; 125% tax pass-through). It also used a higher price elasticity of −0.8, cross price elasticities of 0.1, and income elasticity of 0.3.

c *Source*: Euromonitor International.^8^

d *Source*: Zupanič *et al*.^9^

e These point estimates do not take into account year-to-year variation in sales volume or sampling error when estimating sugar content.

f *Source*: World Health Organization Regional Office for Europe.^7^

g Sugar content for flavored and functional waters was not available. These only accounted for 0.02% of bottled water sales in Ukraine by volume in 2019.

The best-case scenario resulted in a reduction in demand of only 162 metric tons of sugar, largely driven by the small decrease in consumption of carbonates. The worst-case scenario resulted in a reduction in demand of 23 000 metric tons of sugar, again largely driven by carbonates. This amount of surplus sugar could be easily absorbed by the sugar export market, which averaged 449 000 metric tons (186 000–813 000) over 2016/17–2020/21.^6^

In 2019, there was a difference between export and domestic prices of USD 57 per metric ton. The worst-case scenario would result in a USD 1.3 million revenue loss, which was 0.2% of the total sectoral output in 2019. A similar calculation for 2018 when the price gap was higher at USD 134 per metric ton estimate a loss of 0.4% of total sectoral output.

## Discussion

Sugar in SSBs represents only around a tenth of the sugar produced and consumed in Ukraine. In the worst case, implementation of a volume-based SSB tax would result in a reduced demand of 23 000 metric tons of sugar. Sugar output in Ukraine has consistently exceeded domestic consumption for at least the last 20 years and the country has been able to export surpluses.^6^ It is reasonable to expect that the sugar industry could compensate for the estimated small reduction in domestic sales by increasing exports. Due to the import tariff-rate quota regime, however, substituting exports for domestic sales would not compensate for the estimated drop in domestic sales revenues by increased export sales revenues. However, this estimated loss was <0.5% of total sectoral revenue in recent years. Also, as noted for Australia, there could be additional costs for producers to expand export channels or localized adjustments (e.g. mills or refineries with restricted markets) that could merit a small government transition package.^3^

Limitations of this analysis include the use of modeled rather than observed sales reductions, aggregated categories of SSBs, and use of non-local sugar content values. However, increased accuracy in these aspects is unlikely to change our conclusions, given the magnitudes involved.

In conclusion, implementation of a volume-based SSB tax in Ukraine is likely to have a very limited impact on domestic sugar producers. Further research is needed to understand impacts in various contexts and tax structures, for example a tax based on sugar content may result in larger decreases in sugar demand. Investigation of the market dynamics in countries where production and consumption are more aligned and the impact when multiple countries in a trade bloc implement SSB taxes would also be of interest.

## Data Availability

The data underlying the scenarios are from the World Health Organization Regional Office for Europe’s report available at: https://apps.who.int/iris/handle/10665/346226. The data on sugar content are from Zupanič *et al*. (2018) available at: https://doi.org/10.3390/nu10020151. The data underlying the revenue loss estimates are from the State Statistics Committee of Ukraine available at: https://ukrstat.gov.ua. The sales volume data are from Euromonitor International and proprietary.
